# Phage therapy and the public: Increasing awareness essential to widespread use

**DOI:** 10.1371/journal.pone.0285824

**Published:** 2023-05-18

**Authors:** Sophie McCammon, Kirils Makarovs, Susan Banducci, Vicki Gold

**Affiliations:** 1 Living Systems Institute, University of Exeter, Exeter, United Kingdom; 2 Faculty of Health and Life Sciences, University of Exeter, Exeter, United Kingdom; 3 The Department of Social and Political Sciences, Philosophy and Anthropology, University of Exeter, Exeter, United Kingdom; Jaramogi Oginga Odinga University of Science and Technology, KENYA

## Abstract

Today, the antimicrobial resistance (AMR) crisis is shaping a world where previously treatable infections can kill. This has revitalised the development of antibiotic alternatives, such as phage therapy. The therapeutic use of phages, viruses that infect and kill bacteria, was first explored over a century ago. However, most of the Western world abandoned phage therapy in favour of antibiotics. While the technical feasibility of phage therapy has been increasingly investigated in recent years, there has been minimal effort to understand and tackle the social challenges that may hinder its development and implementation. In this study, we assess the UK public’s awareness, acceptance, preferences and opinions regarding phage therapy using a survey, fielded on the Prolific online research platform. The survey contained two embedded experiments: a conjoint and framing experiment (N = 787). We demonstrate that acceptance of phage therapy among the lay public is already moderate, with a mean likelihood of acceptance of 4.71 on a scale of 1 (not at all likely to accept phage therapy) to 7 (very likely to accept phage therapy). However, priming participants to think about novel medicines and antibiotic resistance significantly increases their likelihood of using phage therapy. Moreover, the conjoint experiment reveals that success and side effect rate, treatment duration, and where the medicine has been approved for use has a statistically significant effect on participants’ treatment preferences. Investigations altering the framing of phage therapy, to highlight positive and negative aspects, reveal a higher acceptance of the treatment when described without using perceived harsh words, such as “kill” and “virus”. Combined, this information provides an initial insight into how phage therapy could be developed and introduced in the UK to maximise acceptance rate.

## Introduction

The antimicrobial resistance (AMR) crisis is a global scientific and societal challenge. Antibiotics are becoming increasingly ineffective against previously susceptible bacteria, which will likely force the world into a post-antibiotic era where common infections and minor injuries could kill [1]. In 2019 alone, it was estimated that 1.27 million deaths were attributable directly to bacterial antimicrobial resistance [2], with predictions that as many as 10 million people could die annually from antimicrobial resistance by 2050 [3]. Globally, this has driven renewed interest in alternative treatments such as phage therapy [4]. While public awareness in relation to AMR has been steadily increasing, there is nevertheless a great deal of misunderstanding about the implications and possible solutions [3].

Bacteriophages (or phages) are viruses that infect specific target bacteria [5]. Phage therapy is the administration of phages into a patient to kill the bacterial pathogen, without being able to infect human cells [5]. Unlike antibiotics, phages are biological entities that can only replicate within their specific target bacteria [6]. This high specificity and self-limiting ability means the commensal microbiome of the patient remains intact, resulting in minimal side effects when compared to antibiotic treatment. Consequently, accurate identification of the disease-causing pathogen is required, making phage therapy a personalised medicine [6]. Phages’ natural abundance in the environment also means they are cheaper to produce than conventional antibiotics.

Whilst research into phages has only recently been revitalised in the Western world, phage therapy is no new phenomenon. Phages were discovered independently in 1915 and 1917 by Frederick Twort and Felix d’ Herelle respectively [7, 8]. In subsequent years, phage therapy was used experimentally to treat several bacterial infections, including cholera and dysentery [9, 10]. However, Cold War politics played a key role in the history of phage therapy and, by the 1930s, research became isolated within Eastern Europe and India [11, 12]. This, along with the discovery and subsequent mass production of antibiotics in the post-World War era, led to phage therapy research being mostly abandoned in the West [11, 13]. Instead, phage research was re-focussed into developing tools for biotechnology, yet countries such as Poland and Georgia continued to successfully develop phages as therapeutic treatments [1, 13]. The impending AMR crisis has resulted in a renewed interest in phage therapy research worldwide, with numerous recent success stories finding their way into the public eye [4, 14–17].

Frameworks for phage therapy vary from being non-existent to well-defined. The current regulations surrounding phage therapy mean that it can only be used in compassionate cases in most counties [18]. Achieving global recognition and implementation of phages as a treatment strategy still faces numerous scientific, technological, economic and social challenges. There has been an effort to conduct phage therapy studies that comply to both good manufacturing and clinical practices [19, 20]. However, clinical trials for phage therapies are complex and it is challenging to extrapolate and generalise its safety and effectiveness from small scale trials [21]. In addition, phages are biologically evolving entities and thus do not lend themselves easily to current manufacturing categories and development models imposed on other treatments, such as antibiotics [4, 22]. This constraint, in addition to complexities in patenting natural products, contributes to the lack of private funding for bacteriophage research [4]. Lastly, the development and use of phage therapies must be understood in a socio-political context, where the public and political factors can facilitate or hinder treatments that are successful in trials [4, 23].

The response to medical initiatives highlights how communication of a new treatment to the public can have a profound impact on uptake [24]. For example, recent research on vaccine hesitancy demonstrates how socio-political factors such as communication and media environment, beliefs and attitudes about health and policies are related to vaccine uptake [25]. In this study our aim is to measure the UK public’s opinion of phage therapy as an alternative to antibiotics, providing insights into how phage treatment could be effectively integrated into society with the highest level of acceptance. We hypothesise that the public’s perception of phage therapy treatment is an important component of the socio-political context for the successful development and acceptance of phages as a treatment. Understanding the public awareness surrounding phages and how this relates to the determinants of preferences about this type of treatment is critical knowledge for their long-term development and use. To our knowledge, only one phage therapy opinion survey has been previously published, and this focussed on a specific group of patients with diabetic foot infections, in Scotland [26]. Patient acceptability of phage therapy appeared to be high, with participants expressing a disire for phage therapy to be offered as an alternative treatment option [26]. We explore how these results compare to the opinions of the lay UK public.

Our method of measurement is a public opinion survey with embedded experiments. These survey experiments are useful for understanding effects of wording and of treatment attributes that may be important determinants of preferences. Theories of survey response do not posit that individuals have a store of attitudes about all potential issues. Rather, their responses to these survey questions are viewed as “constructed preferences” [27]. Influential psychological models of the survey response suggest individuals draw from a range of salient considerations that are immediately available to them (“off the top of their head”) [27]. The news of the day, experiences from their lives, the wording of the questions themselves can affect the salient considerations that respondents draw on to formulate an answer to the questions. We identify numerous attributes that influence participants’ preference towards antibiotic-alternatives, including, side effect and success rate, duration of treatment and where it has been approved for use. Moreover, describing phage therapy using perceived harsh words, such as “virus” and “kill”, is shown to significantly decrease acceptance of the treatment. However, exposure to only a limited amount of information regarding antibiotic alternatives appears to greatly increase public acceptance of phage therapy.

## Data and methods

There are three main components to the data we report: a conjoint (discrete choice) experiment, a framing experiment, and responses to an open-ended question. We provide details about the methods for data collection in the following sections. The study was approved by and adheres to the regulations of the University of Exeter’s Faculty of Health and Life Sciences Research Ethics Committee. Consent to participate in the survey was informed by the provision of an approved participant information sheet and completion of an online (written) consent form at the beginning of the survey. If consent was not provided, participants could not access the survey.

Qualtrics was used to design an online survey containing four main sections. The first section comprised of socio-demographic questions, including age, gender and educational level. The participants’ health-literacy of current medicine-related news, such as antibiotic resistance, phage therapy and conspiracy theories, was also assessed. The second and third sections contained the conjoint and framing experiments described below. The final section comprised of an open-ended question asking for the participants’ thoughts on phage therapy and whether it could provide an alternative to antibiotics.

We describe below the use of an academic researchers’ workshop to inform the variables to be included in the conjoint and framing experiments. The academics’ meeting was attended by 23 members of the University of Exeter’s Life Science community, on 11 November 2021.

### Conjoint experiment

Conjoint experiments, also known as discrete choice experiments (DCEs), are used to measure the value people place on different attributes of a service or product [28]. This technique is used extensively in healthcare, specifically to assess the characteristics of various medicines influencing patients’ treatment preferences [28–30]. In this study a conjoint experiment was used to evaluate the general public’s preferences regarding various attributes relevant to both phage therapy and antibiotics. The main advantage of the conjoint experimental design over classical survey experiments is that it allows accounting for multiple treatments and delineating their causal effects. However, this study had some limitations. The number of displayed attributes has to be restricted to no more than six or seven. Exceeding this number would entail an increased cognitive burden put on the respondents, leading to cognitive shortcuts in evaluating profiles and making choices [31]. There are also certain restrictions for the number of levels per attribute, as the more levels are inspected, the larger the sample size should be to detect the statistically significant effects. Another limitation of the conjoint experimental design is that it is suitable for studying only those types of behaviours and attitudes that can be operationalised in the form of discrete binary choice or ranking questions [32]. More generally, survey experiments of any kind are criticised for having a limited ability to shed light on real-world behaviour due to the artificial nature of the experimental setting that the respondents are put in [33]. However, as Hainmueller *et al*. show, the results produced in the conjoint experiment can closely approximate the real-world behavioural benchmark [34].

Our strategy for determining the attributes and levels for the conjoint experiment drew on both an academic workshop and a thorough review of research. Our review of relevant literature, exploring characteristics of treatments that had been used in prior conjoint studies, identified a list of 17 attributes, and their associated levels [30, 35–37]. The attributes included factors such as magnitude of treatment benefit, contribution to antibiotic resistance and severity of treatment side effects [30, 35–37]. Some attributes specific to phage therapy were also included [38]. Based on this review, we selected 12 attributes that were most relevant for treatment of antibiotic resistant infections to present to our group of academics, during the workshop. The participants at the workshop were given two scenarios; in the first they presented with a minor infection, and in the second they presented with an infection that did not respond well to antibiotics for three months. In each scenario, the group ranked the selected attributes based on their importance in deciding whether to accept a treatment or not. Success rate and severity of side effects were the highest ranked attributes in both scenarios, with contribution to antibiotic resistance appearing to only be of importance when the infection was minor. From these responses, 5 attributes were selected; 3 are relevant to the general treatment of drug resistant infections, and 2 attributes specific to phage therapy. Table 1 defines the selected attributes, along with their associated levels.

**Table 1:** List of selected attributes and levels, with definitions.

Attribute	Definition	Levels
Side effects	All medicines may have side effects, including nausea, headache and tiredness. Here, it is measured how many people will get mild side-effects from the treatment.	1% (1 in 100) people using this therapy get side effects. 5% (5 in 100) people using this therapy get side effects. 10% (10 in 100) people using this therapy get side effects. 20% (20 in 100) people using this therapy get side effects.
Success rate of Therapy	A medical treatment can fail to resolve an infection for many reasons, meaning you have to receive another course. Success rate measures how many people will need no further treatment after the original course.	20% (20 out of 100) people need no further treatment. 50% (50 out of 100) people need no further treatment. 80% (15 out of 100) people need no further treatment.
Duration of Treatment	Medicines will need to be taken for different amounts of time to be effective. Here, the medicine must be taken 3 times a day throughout the specified treatment period.	Must take treatment for 2 weeks but can end earlier if infection clears. Must take treatment for at least 3 days but can stop when infection clears. Must take treatment for 2 weeks even if infection clears.
Type of Treatment	Combinations of various phage-types along with other treatments can be taken. In this case, all the options are administered in an identical manner.	One type of phage only. Combination of different phages. Combination of phage and antibiotic. A phage protein.
Approved for use	Regulations regarding phage therapy vary world-wide, with it being approved in only some countries.	Approved in a few countries, like Georgia. Approved in a few countries, like Belgium. Used as a therapy of last resort in the UK.

Side effects, success rate and treatment duration are general attributes of medical treatments, while type of phage treatment and where the treatment is approved for use include levels associated specifically with phage therapy.

**Fig 1:**
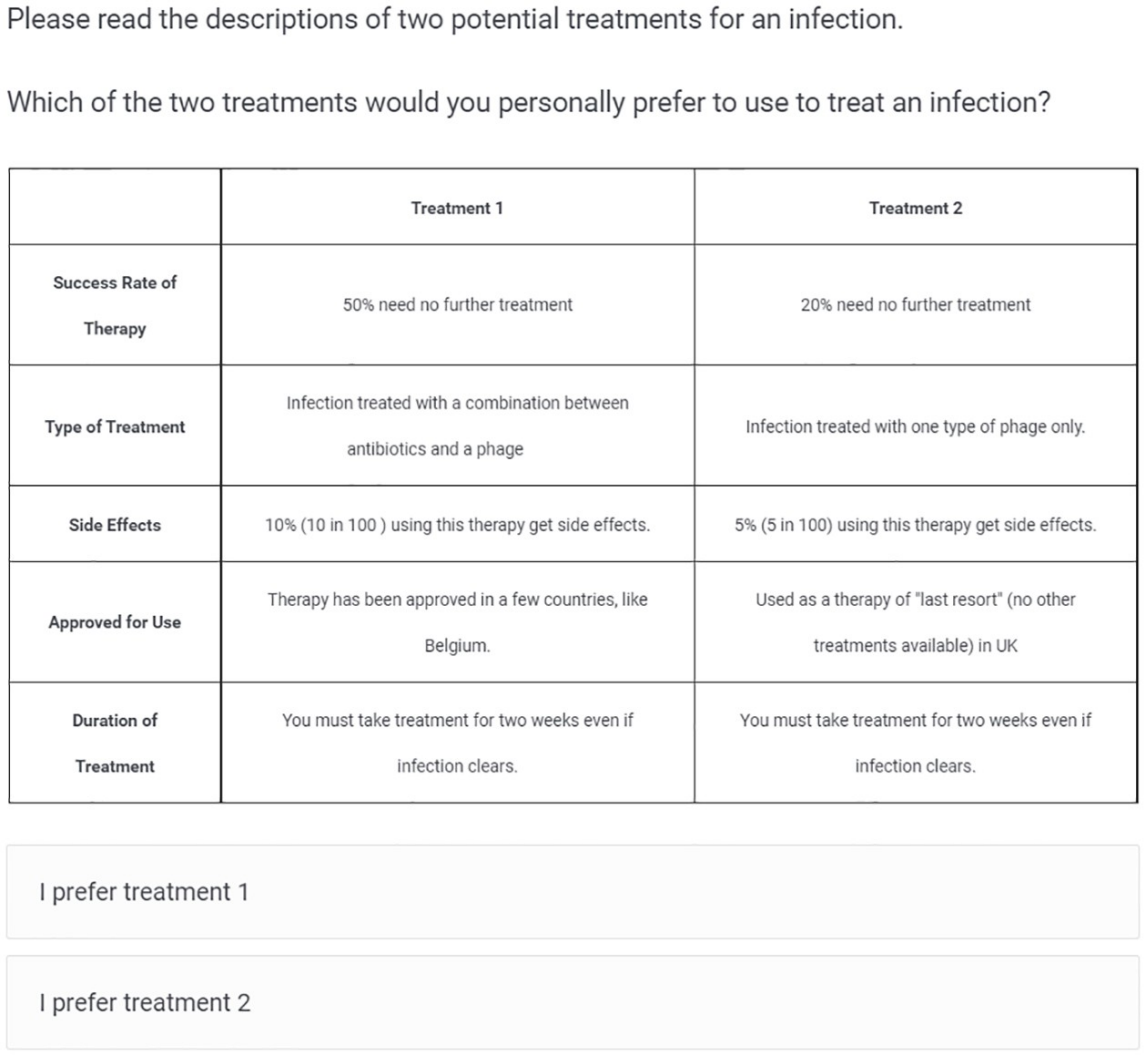
Conjoint experiment: Example of screen seen by study participants. Attribute levels were randomly assigned to create two hypothetical treatments. Participants were asked to express a preference for Treatment 1 or Treatment 2 and rank the likelihood of use of each treatment on a scale of 1 (not likely al all) to 10 (very likely).

These attributes and levels from Table 1 are used to construct two hypothetical treatments that are presented as a set of attributes, with the levels of each attribute varying between the two options (Fig 1). By asking respondents to express a preference, we can determine the influence each attribute has on their choice. After respondents made a choice to which treatment they prefer, they were next asked to evaluate, on a scale from 1 to 10, where 1 indicates “not at all likely” and 10 indicates “very likely”, their likelihood of using each treatment. Therefore, we have both their discrete preferences (treatment 1 or 2) and a ranking for each treatment on likelihood of use, allowing us to capture “discrete preferences” and “attitudes” about the treatments [39]. We use both outcomes in our analysis to understand which attributes are most influential in choosing alternative treatments. Each participant was presented with 5 hypothetical choice sets.

### Framing experiment

We also employed a second type of experiment–a framing experiment–where we randomly assigned respondents to receive one of four descriptions of phage therapy. Framing experiments have demonstrated that even very small changes in how issues are presented to the public can alter their opinions [40]. It has long been recognised that framing of attributes as gains or losses has an impact on perceptions and choices, though context appears to be important and there is a lack of consistent evidence on this type of framing in patient and consumer choices in health [41, 42]. We varied the framing to highlight different aspects associated with phage therapy to assess how this effects the participants’ likelihood of treatment acceptance. This allowed us to assess which aspects of phage therapy are rendered more or less salient in the survey question [41].

Because phage therapy is not currently a widely available treatment for infections in the UK, we opt to vary the emphasis on negative (“kill cells”) and positive (“friendly viruses”) definitions. We draw on the extant literature, a review of news media stories about phage therapy and our expert workshop to determine the different descriptions of phage therapy we would present in the framing experiment [14–17]. From this review, four different descriptions of phage therapy were created:

1. Phage therapy uses live viruses to kill cells
2. Phage therapy uses viruses to selectively treat infections
3. Phage therapy uses natural bacterial predators to treat infections
4. Phage therapy uses friendly viruses to treat infections

These four descriptions were presented to all attendees of the academic workshop. The likelihood of the academic group accepting treatment based on the descriptions varied between all four options. Hence, we decided to randomly assign one of these definitions to each survey participant.

### Ordering

Within the survey, we randomised the framing and conjoint experiments to capture any impact of ordering of the two experiments. Our framing experiments allows us to capture how one dimension of phage therapy–how it is defined–may alter responses, but conjoint experiments allow us to vary several potentially important attributes about the treatment. Our concern with the ordering of the two experiments was whether the description of phage therapy the participants received in the framing experiment would “prime” respondents to think about phage therapy in a particular way. According to Fiske and Taylor, priming describes the cognitive process whereby “recently and frequently activated ideas come to mind more easily than ideas that have not been activated” [43]. If exposed to the framing experiment first, respondents may access these considerations more readily for the conjoint experiment [44]. We examine and discuss any ordering effects of the experiments in the results section below.

### Study participants and period of the study

We conducted the two experiments in an online survey using a panel of participants from Prolific. In Prolific, we designated that our final sample should be representative of the British adult population. Prolific ensures the representativity of the sample by stratifying it by age, sex, and ethnicity according to the census data of the UK Office of National Statistics [45]. Compared to other online participant recruitment platforms such as MTurk, Prolific shows higher overall data quality, and provides a more diverse population of participants [46].

The survey was fielded 14–15 December 2021 using the Prolific online research platform, with a total sample size of 832. Respondents had an average completion time of 10.5 minutes. Respondents who completed the survey in less than 4 minutes were discarded (n = 42) to preserve data integrity [47, 48]. This left an effective sample size of 787. Of this adjusted population, 51.6% were women and 47.9% were men. The mean age of the participants was 47.2 years, with a standard deviation of 15.5. For further sociodemographic information, refer to the data in S1 Appendix.

### Statistical analysis

For our analysis of the conjoint experiment, we used the cregg package by Leeper to calculate both the average marginal component effects (AMCE) and the marginal means [49]. The AMCE can be interpreted as indicators of “causal effect” coefficients and the marginal means gives the overall favourability of an attribute with the mean support (0 to 1). Marginal means, then, can provide a descriptive account of the attributes in our sample and give an indication of the mean outcome of an attribute, such that means with averages above the midpoint indicate a positive effect on infection treatment preference and below the midpoint indicates a negative effect. Marginal means are also the preferred method for comparing sub-group differences due to the sensitivity of AMCE to the choice of baseline [50]. The baseline level was the default generated by the estimation procedure [49].

## Results

### Factors influencing treatment preferences

**Fig 2:**
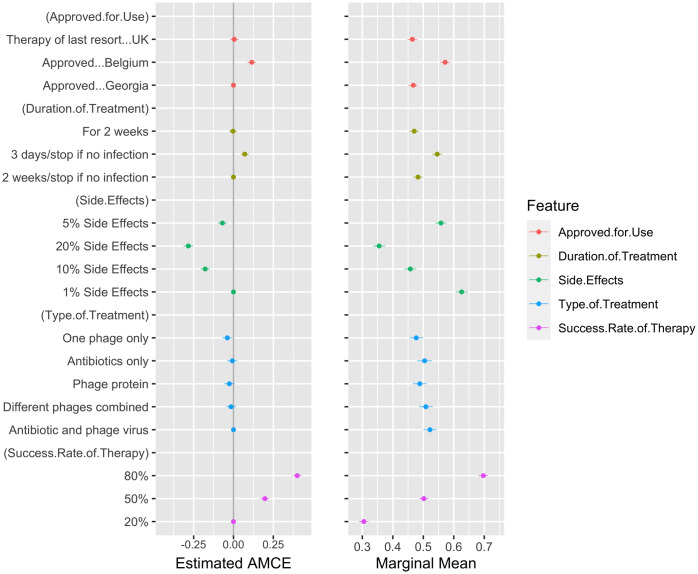
AMCE and marginal means for discrete preferences. For our analysis of the conjoint experiment, we calculated both the AMCE (left panel) and the marginal means (right panel). The AMCE can be interpreted as indicators of “causal effect” coefficients, showing the average conditional effects for each attribute relative to the baseline. The marginal mean gives the overall favourability of an attribute with the mean support (0 to 1), where above 0.5 indicates a positive effect of the attribute on treatment preference, and below 0.5 indicates a negative effect. The figure plots the estimated values and the 95% confidence intervals for these estimates. These exact values are available in S2 Appendix. When confidence intervals do not overlap, we take this to suggest that the estimates are significantly different from one another statistically.

Fig 2 (left panel) shows the AMCE for each attribute relative to a baseline level. Considering this, along with the marginal means (Fig 2, right panel), success rate was the most influential attribute when deciding treatment preference, followed by side effect rate. Increasing the success rate from 20% of people needing no further treatment to 50% increases the participants preference towards a treatment by 0.19. Increasing this to 80% results in a further 0.2 increase in treatment preference. On the other hand, moving from 1% side effects to 20% side effects reduces support by over 0.25 (-0.27).

Knowledge of approval influences preferences and there is a positive preference towards Western European countries. If the treatment was stated to be approved in Belgium (relative to Georgia) this increased preference, whereas treatments approved as a “last resort in the UK” did not significantly increase choice of that option. Treatments with shorter administration periods were also preferred; however, for the 2-week treatment courses, there was no difference between those that could be stopped early or those that had to be taken for the complete duration (like current antibiotics).

The type of treatment seemed to have little effect on the participants’ treatment preference. This attribute asked respondents to consider the different ways in which phages can be used to treat infections. We expect that this required understanding of how antibiotics and phage therapies work. Given very few respondents in the sample were aware of phage therapy, the lack of effect for this attribute may have resulted from a lack of awareness.

**Fig 3:**
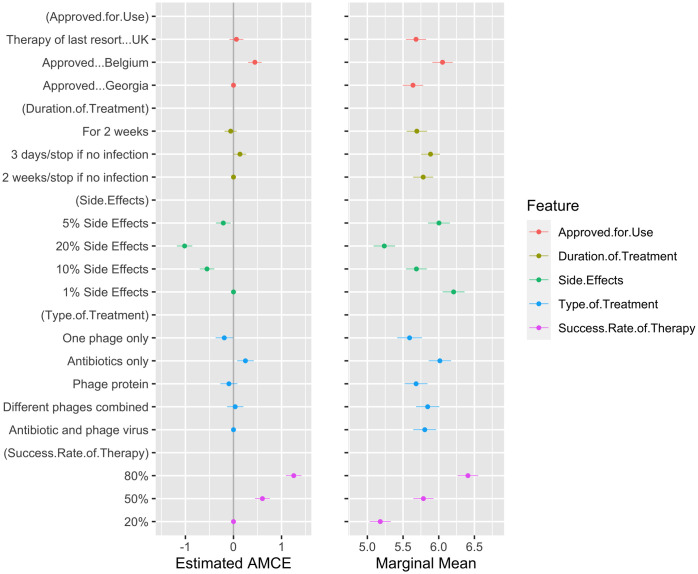
AMCE and marginal means for preference rankings. The ACME (left panel) and marginal means (right panel) were calculated for the ranked preferences of the infection treatments presented in the conjoint experiment. AMCE assesses the marginal effect of attributes on attitudinal measures whereas the marginal means gives the predicted mean ranking for each attribute level. The figure plots the estimated values and the 95% confidence intervals for these estimates. These exact values are available in S2 Appendix. When confidence intervals do not overlap, we take this to suggest that the estimates are significantly different from one another statistically.

After determining the respondent’s discrete preferences, we asked them to evaluate, on a scale from 1 to 10, where 1 indicates “not at all likely” and 10 indicates “very likely”, their likelihood of using the hypothetical infection treatments presented in the conjoint experiment. The results for this analysis are shown in Fig 3. Ranked preferences allow us to examine the impact of attributes on attitudinal measures [39]. The results in Fig 3 confirm the discrete choice analysis in Fig 2. Compared to the analysis of discrete preferences, the rankings, as indications of evaluative attitudes about the hypothetical infection treatments, confirm the importance of high success rates, approval in Belgium and lower side effects for more positive evaluations of the alternative treatments. There is a slight difference for the duration of treatment between ranked preferences and discrete preferences but, in general, there is still preference for shorter duration.

### Framing and ordering effect on phage therapy acceptance

**Fig 4:**
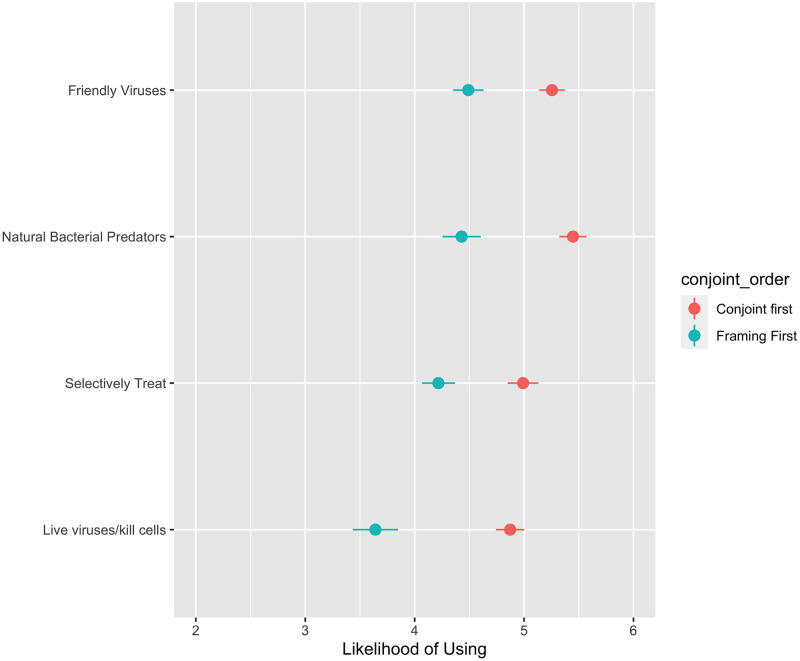
Framing experiment responses. The left panel shows the average “likelihood of use” across the four framing conditions: (1) Phage therapy uses live viruses to kill cells, (2) Phage therapy uses viruses to selectively treat infections, (3) Phage therapy uses natural bacterial predators to treat infections, (4) Phage therapy uses friendly viruses to treat infections, where 1 is “not likely at all” and 7 is “very likely”. The figure compares the averages from those who completed the conjoint experiment before the framing experiment (red) and those who completed the framing experiment before the conjoint experiment (blue). The figure plots the estimated values and the 95% confidence intervals for these estimates. These exact values are available in S2 Appendix. When confidence intervals do not overlap, we take this to suggest that the estimates are significantly different from one another statistically.

Regarding the respondents’ acceptance of phage therapy based on the description provided, there appeared to be very little framing effect (Fig 4). Overall, there is moderately high support for all phage therapy descriptions, with a mean likelihood of acceptance of 4.71 on a scale of 1 (not at all likely to accept phage therapy) to 7 (very likely to accept phage therapy). There are small differences between each description, with lower levels of likelihood of use for respondents who were shown the definition of phage therapy that could be perceived as the most extreme (“live viruses being used to kill cells”). There are only significantly lower levels of acceptance when phage therapy is described as using “live viruses to kill cells”, compared to “natural bacterial predators to treat infections”. The framing which gained highest support is the only description to not contain the word “virus”.

The most significant finding from this experiment was related to the ordering of the framing experiment relative to the conjoint experiment. Being exposed to the conjoint experiment before the framing experiment increased the likelihood of phage therapy use across the board (Fig 4). The relative difference in the level of acceptance between the four framing options did not differ by ordering of experiment. Thus, there appears to be an overall information or priming effect; those who had recent exposure to information about antibiotic resistance and alternative treatments from completing the conjoint first were more willing to accept phage therapy in the framing experiment.

**Fig 5:**
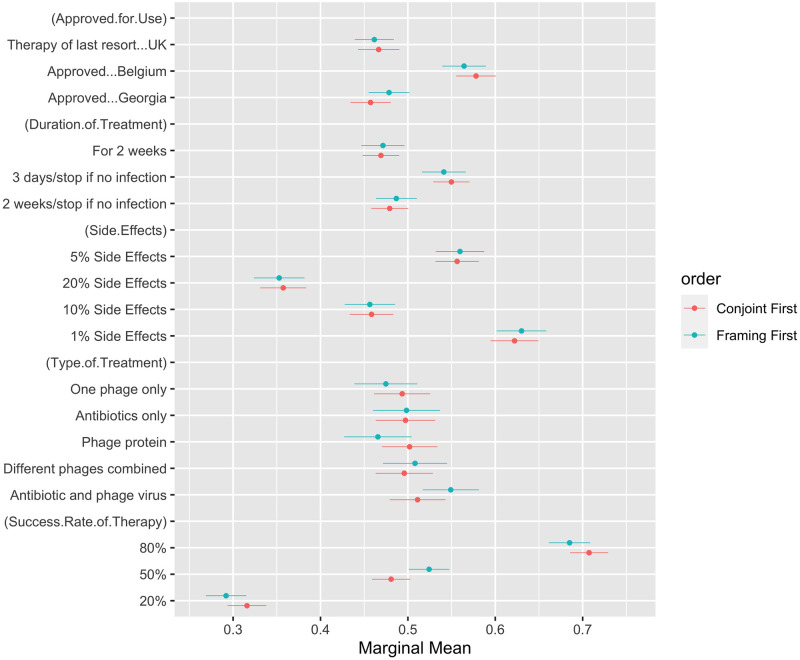
Marginal means by ordering of experiments. The marginal means give the overall favourability of an attribute with the mean support (0 to 1), where above 0.5 indicates a positive effect of the attribute on treatment preference, and below 0.5 indicates a negative effect. This figure compares the marginal means from those who completed the conjoint experiment before the framing experiment (red) and those who completed the framing experiment before the conjoint experiment (blue). The figure plots the estimated values and the 95% confidence intervals for these estimates. These exact values are available in S2 Appendix. When confidence intervals do not overlap, we take this to suggest that the estimates are significantly different from one another statistically.

We had no hypothesis about the impact of the ordering of the experiments on the outcomes for the conjoint experiment. Fig 5 confirms there is no significant difference in the participants’ responses to the conjoint experiment, whether they received the framing experiment before or after. In our case, being exposed to information about phage therapy before the conjoint experiment did not significantly alter the conditional effects of the characteristics of general antibiotic-alternative treatments.

### Open ended responses

**Fig 6:**
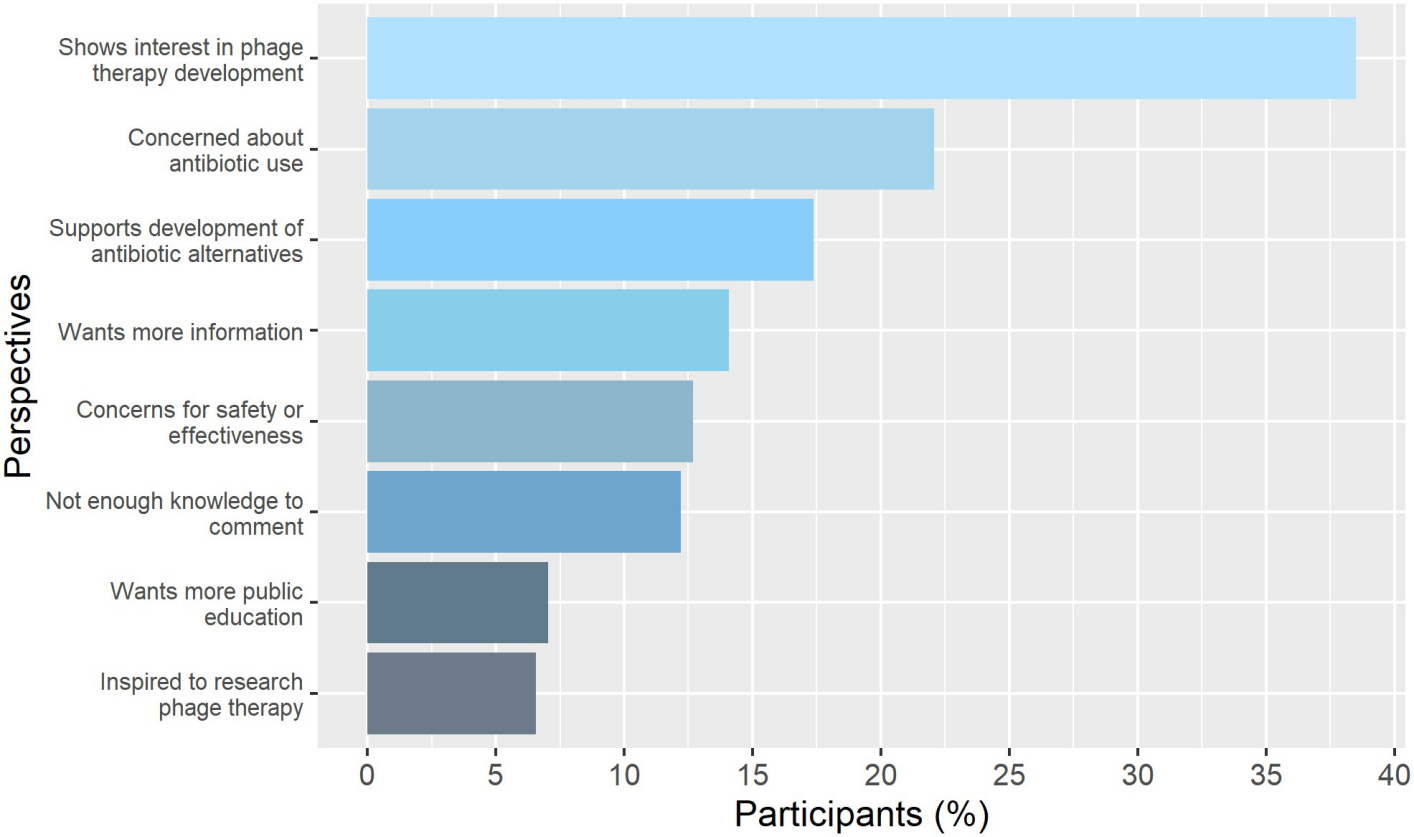
Open answer responses. 267 Participants left responses to the open question; 213 of these were relevant to the question and categorised into the 5 answer categories shown in the graph. Each response was included in all relevant answer categories, as shown in S2 Appendix.

From the 787 participants who completed the survey, 213 left relevant written answers in response to the open-ended statement: “We would be interested in hearing any additional thoughts you have on phage therapy and whether they could provide an alternative to antibiotics in treating infections” (Fig 6). Of the 213 responses, 38.50% showed a specific interest in phage therapy development, while a further 17.37% supported the development of antibiotic alternatives generally. 6.57% were inspired to conduct their own research and 7.04% wanted information to be more publicly available.

## Discussion

While phage therapy has existed for over a century, the success and widespread availability of antibiotics, alongside political and socio-economic factors, has stalled its development. However, in the age of the AMR crisis, it is vital to make alternatives technologically, economically and socially feasible. Public acceptance of a treatment is a key factor in its success and uptake, and assessment of the public opinion surrounding phage therapy has been previously neglected [24].

Congruent to previous reports, our lay respondents appeared to have a high awareness of antibiotic resistance [26, 51]; 92% had heard of antibiotic resistance, but only 13% reported that they had heard about phage therapy prior to the survey (N = 787). While phage therapy remains poorly understood by the UK public, the responses to our open-ended question, along with our framing experiment, suggest there is extensive acceptance and support for its development. Our framing experiment also suggested exposure to only very limited information about antibiotic resistance and alternative treatments to antibiotics greatly increases the public acceptance of phage therapy. There was a significantly greater level of phage therapy acceptance if respondents had completed the conjoint experiment before the framing experiment. The information about antibiotic resistance in the conjoint experiment introduction statement, along with the general and phage-specific attributes, may have primed participants to access considerations regarding this novel treatment more readily [44]. Previous studies conclude the apparent support for development of antibiotic alternatives may stem from concerns surrounding the antibiotic resistance crisis [52, 53]. This implies that public education focused on antibiotic resistance has been successful and there is a positive effect of public awareness of the discussion on alternative disease treatments on their rates of acceptance.

Participants also expressed a desire for there to be increased public education on phage therapy; 14 respondents even stated they were inspired to research this topic after completing the survey. This effect has been seen previously in the UK. During May 2019, there was a peak in Google searches for “Phage Therapy”, possibly relating to the release of news articles describing the success of phages in controlling an aggressive *Mycobacterium* infection in a 16-year-old British patient [14, 54, 55]. This suggests if phage therapy continues to become more prevalent in the news, there will be an increased demand for accessible education, to limit negative speculation. To meet this, publications from countries that have been utilising phage therapy successfully for decades could be translated and adapted for the UK and global population. Involving the public, specifically children, in phage collection is also a promising form of public education. For example, the Citizen Science Phage Library (https://citizenphage.com) characterises phages collected by the public from the environment [56]. By targeting schools and science fairs for their recruitment of “phage hunters”, there is the potential for phage therapy to become common knowledge amongst a generation who may need to utilise antibiotic alternatives. Not only does this expose the public to phages in a positive, engaging manner, but due to phages’ abundance in the natural environment, this approach provides a low-cost, replicable template for accelerating the development of phage libraries globally [57].

Due to the fact phage therapy awareness is low, there needs to be consideration of how bacteriophages can be comprehensibly presented to general society. Where awareness of an issue is low, the topic is not salient in the news and there is little lived experience, the cognitive demand on the population may be particularly high, introducing error into understanding and response [27]. In these situations, how the treatment is communicated to the public can have a profound impact on uptake [25]. For example, studies investigating broadcasting relating to vaccines shows that media reporting on vaccine safety can influence public perceptions, and ultimately acceptance [24]. Our framing experiment shows there is highest acceptance of phage therapy when it is described without using the word “virus”, instead using “natural bacterial predator”. This information may be particularly relevant in the wake of the viral COVID-19 pandemic. Similarly, there is a positive preference towards therapies publicised as approved for use in a Western European country, compared to an Eastern European country. This Western-European bias for medical treatments has been illustrated previously, most notably with vaccines [58]. Combined, this information may be used to influence phage marketing and advertisement to maximise acceptance.

Our conjoint experiment showed that shorter treatment durations are preferred. For antibiotics, treatment courses can vary dramatically, but in 2014, a 6–7-day antibiotic course was most commonly prescribed for acute infections [59]. In the limited phage therapy clinical studies reported, there is extreme variation in treatment duration, ranging from a single dose, up to 32 weeks [60]. Further research needs to be conducted into phage therapy treatment in relevant clinical settings to determine specific treatment regimes. There was also a preference for lower side effect rates. For example, moving from a 1% side effect rate to a 20% side effect rate reduces treatment preference by over 0.25. An advantage of phage therapy is its high specificity for the bacterial pathogen [6]. This means phages have limited interaction with the beneficial bacteria in the human body, which in most cases result in minimal side effects [6]. In contrast, antibiotic treatment can cause dysbiosis, resulting in a plethora of undesirable reactions such as antibiotic-associated diarrhoea and even long-term immunological disorders [61]. Emphasising this through education and marketing may increase public acceptance of phage therapy, potentially making it preferable to antibiotics.

## Conclusion

Even though phage therapy may be some years away from routine clinical use in the UK, increasing pressures from the AMR crisis require evaluation of the UK public’s acceptance of alternative treatments. The public shows a high awareness of antibiotic resistance, which appears to result in extensive support for development of novel therapeutics. The findings suggest exposure to only a very limited amount of information about antibiotic resistance and alternative medicines significantly increases acceptance of phage therapy, possibly through the priming effect [44]. Additionally, the public desire for increased education is apparent. Expanding schemes which are interactively involving children in phage research not only generates excitement for the therapy now, but also promotes awareness in the generation likely to be treated with antibiotic alternatives [56]. The wording used to advertise phage therapy successfully also seems to be extremely important; using alternative descriptions to words perceived as more severe, such as “kill” and “virus”, along with highlighting that phage therapy is approved for use in specific countries, appears to increase public acceptance.

Further research exploring the influence other attributes of alternative medicines to antibiotics have on treatment preference needs to be explored. These attributes include magnitude of treatment benefit, phage development (naturally occurring or genetically modified) and how the treatment is administered. Comparison of the UK public’s acceptance of phage therapy to the public opinion of countries that are routinely using phage therapy, may also be insightful. This may expose strategies which resulted in successful implementation and allow us to anticipate deep-rooted concerns that may hinder phage therapy acceptance, even after years of routine clinical use. Previous research has highlighted the apparent hesitation of health and scientific professionals towards phage therapy implementation [4]. Case studies suggest this may arise from the lack of phage availability and ill-suited regulatory infrastructure [4]. However, even though these professionals are likely to be significantly influential in the acceptance and uptake of phage therapy, to our knowledge, there is yet to be a large-scale assessment of the UK’s medical professionals’ opinions. Hence, evaluation of their acceptance, concerns and the factors that influence their prescribing preferences is an essential next step.

## Supporting information

S1 Appendix Sociodemographic characteristics. (PNG)

S2 Appendix Conjoint experiment, framing experiment and open answer statistical analysis data. (XLSX)
