# Supplementary figures and images for: Phage therapy and the public: Increasing awareness essential to widespread use
Source: PLoS One. 2023 May 18;18(5):e0285824. doi: 10.1371/journal.pone.0285824 (PMC10194857; doi:10.1371/journal.pone.0285824)

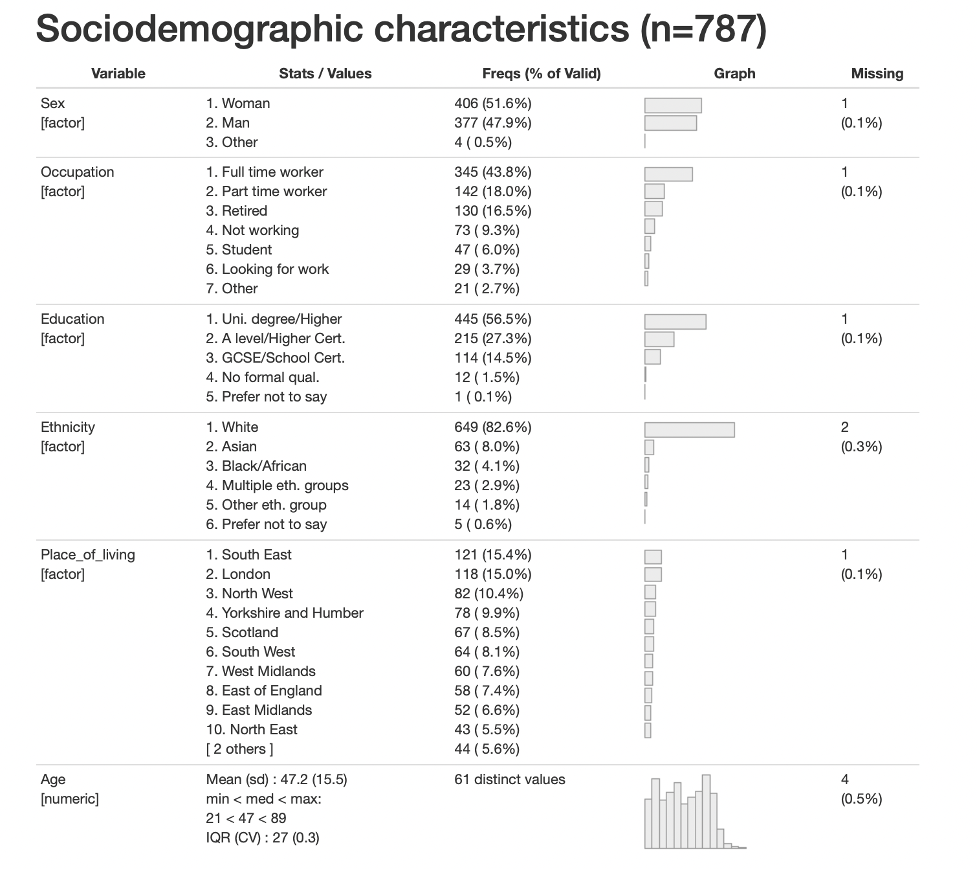

Supplement: S1 Appendix — (PNG) [file pone.0285824.s001.png]
